# Epigenetic Plasticity in Triple-Negative Breast Cancer: Mechanisms of Therapy Resistance, Biomarkers, and Therapeutic Vulnerabilities

**DOI:** 10.3390/biomedicines14092013

**Published:** 2026-09-08

**Authors:** Abdel Raman Alaa, Salma A. B. El-Din, Mohannad A. Farrag, Youssef Ahmed, Mohamed E. Abdel Aziz, Shaimaa Abdel-Ghany, Borros Arneth, Hussein Sabit

**Affiliations:** 1 Department of Medical Biotechnology, College of Biotechnology, Misr University for Science and Technology, Giza P.O. Box 77, Egypt; 200025826@must.edu.eg (A.R.A.); 200040263@must.edu.eg (M.A.F.); 200047168@must.edu.eg (Y.A.); mohamed.elminiawy@must.edu.eg (M.E.A.A.);; 2 Institute of Laboratory Medicine and Pathobiochemistry, Molecular Diagnostics, University Hospital of the Universities of Giessen and Marburg UKGM, Philipps University Marburg, Baldingerstr 1, 35043 Marburg, Germany; 3 Institute of Laboratory Medicine and Pathobiochemistry, Molecular Diagnostics, University Hospital of the Universities of Giessen and Marburg UKGM, Justus Liebig University Giessen, Feulgenstr. 12, 35392 Giessen, Germany

**Keywords:** triple-negative breast cancer, epigenetic plasticity, therapy resistance, epigenetic biomarkers, epigenetic therapy

## Abstract

Triple-negative breast cancer (TNBC) is an aggressive and clinically heterogeneous breast cancer subtype characterized by the absence of estrogen receptor, progesterone receptor, and HER2 overexpression, limited targeted treatment options, early relapse, and frequent development of therapy resistance. Although TNBC often shows initial sensitivity to chemotherapy, durable responses are commonly undermined by the emergence of adaptive resistant cell states rather than solely by fixed genetic mutations. This review synthesizes the role of epigenetic plasticity as a central mechanism that enables TNBC cells to dynamically reprogram transcriptional identity, survive therapeutic stress, and transition between epithelial, mesenchymal, stem-like, immune-evasive, and drug-tolerant persister phenotypes. Key epigenetic mechanisms include aberrant DNA methylation, histone acetylation and methylation, BET/BRD4-dependent transcriptional regulation, EZH2-mediated repression, SWI/SNF-dependent chromatin remodeling, non-coding RNA networks, and three-dimensional genome reorganization. These processes regulate tumor suppressor silencing, DNA-damage repair, epithelial–mesenchymal plasticity, cancer stem-cell maintenance, metabolic adaptation, immune-checkpoint regulation, and minimal residual disease. The review also highlights the translational relevance of epigenetic biomarkers, including DNA methylation signatures, circulating epigenetic markers, chromatin-accessibility profiles, and single-cell epigenomic approaches for diagnosis, prognosis, therapy prediction, and monitoring resistance evolution. Finally, therapeutic strategies targeting epigenetic plasticity are discussed, including DNMT, HDAC, BET, EZH2, KDM, and LSD1 inhibitors, with emphasis on rational combination approaches involving chemotherapy, PARP inhibitors, immunotherapy, and metabolic targeting. Overall, epigenetic plasticity represents both a major driver of TNBC resistance and a therapeutically exploitable vulnerability, provided those future strategies account for tumor heterogeneity, adaptive cell-state transitions, biomarker-guided patient selection, and combination-based treatment design.

## 1. Introduction

Triple-negative breast cancer (TNBC) represents an aggressive and clinically diverse subtype of breast cancer. It is characterized by the absence of estrogen receptor (ER) and progesterone receptor (PR) expression, as well as a negative HER2 status, which is determined by the lack of HER2 protein overexpression and/or the absence of ERBB2 gene amplification [1,2]. Because TNBC lacks the major receptor targets used in endocrine and HER2-directed therapy, systemic treatment has historically relied heavily on chemotherapy, with additional benefit from immunotherapy, PARP inhibition in selected homologous-recombination-deficient tumors, and emerging molecularly guided strategies. Although many TNBC tumors initially respond to chemotherapy, durable clinical benefit is frequently limited by early relapse, metastatic progression, and the emergence of therapy-resistant residual disease [3].

Epigenetic plasticity provides a mechanistic framework for understanding these adaptive transitions. In TNBC, reversible remodeling of DNA methylation, histone modifications, chromatin accessibility, chromatin-reader activity, non-coding RNA networks, and three-dimensional genome organization can alter transcriptional identity without changing the underlying DNA sequence [4,5]. These processes allow tumor cells to switch between epithelial, mesenchymal, stem-like, immune-evasive, metabolically rewired, and drug-tolerant persister phenotypes. Therefore, epigenetic plasticity should be viewed as a central bridge connecting TNBC heterogeneity, minimal residual disease, treatment escape, immune resistance, and recurrence.

This critical review examines how reversible epigenetic states enable treatment adaptation in triple-negative breast cancer. We distinguish molecular regulators of chromatin plasticity from the resistant phenotypes they support, evaluate the strength of evidence linking these mechanisms to chemotherapy, PARP-inhibitor and immune-checkpoint resistance, and rank therapeutic vulnerabilities according to clinical maturity. Unlike recent reviews that broadly catalog TNBC epigenetics, our emphasis is the temporal transition from treatment-naïve cells to drug-tolerant residual states, the biomarkers capable of tracking that transition, and the evidence required to convert epigenetic targets into biomarker-guided combinations.

### Literature-Search Approach

We searched PubMed/MEDLINE, Scopus, and Web of Science for English-language studies published from database inception through 28 March 2026. Search terms combined (“triple-negative breast cancer” OR TNBC) with (“epigenetic plasticity” OR “DNA methylation” OR histone OR chromatin OR “non-coding RNA” OR persister OR “drug resistance”) and, for translational studies, (biomarker OR trial OR therapy). Reference lists of relevant reviews were screened to identify additional primary studies. Priority was given to human TNBC cohorts, prospective clinical studies, and functional TNBC models. Evidence from other breast cancer subtypes, other malignancies, or non-cancer systems was included only when TNBC-specific evidence was unavailable and was explicitly identified as indirect evidence.

## 2. Molecular Mechanisms of Epigenetic Plasticity

Epigenetic plasticity in TNBC is driven by multiple mechanisms, each of which either enhances tumor adaptability and survival or creates vulnerabilities that may be therapeutically exploited. Understanding these underlying molecular mechanisms and distinguishing between them and epigenetic phenomena is the key to developing new meaningful approaches in the field of epigenetic therapy [6].

### 2.1. DNA Methylation Dynamics

DNA methylation is a key epigenetic regulator of gene expression. It involves the addition of methyl groups to CpG sites without altering the underlying DNA sequence and is regulated by DNA methyltransferases (DNMTs) [7]. Differentially methylated regions can be used to compare methylation states associated with different biological phenotypes [8]. Aberrant hypermethylation or hypomethylation contributes to many diseases, including cancer [7]. BRCA1 dysfunction in TNBC may result from either pathogenic sequence variants or epigenetic silencing. Promoter hypermethylation can reduce BRCA1 expression in tumors without a BRCA1 mutation; however, reported prevalence estimates vary across cohorts and assays [9]. Global or focal hypomethylation can activate repetitive elements and oncogenic regulatory regions, thereby contributing to genomic instability [10].

Methyl-CpG binding domain protein 2 (MBD2) sequencing analysis identified ATP1A1 hypomethylation in TNBC. ATP1A1 encodes the alpha-1 catalytic subunit of the Na^+^/K^+^-transporting ATPase, a membrane pump involved in maintaining cellular ion homeostasis and regulating signaling pathways that influence cell survival and proliferation. ATP1A1 hypomethylation and high expression were associated with poor survival, while ATP1A1 knockdown reduced the viability and tumor-sphere formation of TNBC cells, supporting its potential prognostic and therapeutic relevance [11]. CHST2, which encodes carbohydrate sulfotransferase 2, has also been reported to be aberrantly upregulated in TNBC as a consequence of promoter hypomethylation. CHST2 encodes an enzyme involved in carbohydrate sulfation, a modification process that regulates extracellular matrix components and cell–matrix interactions, thereby potentially influencing tumor migration and invasion. Elevated CHST2 expression was associated with aggressive clinicopathological characteristics, and functional analyses showed that CHST2 promotes TNBC cell migration and invasion [12]. In locally advanced breast cancer, neoadjuvant chemotherapy was associated with changes in DNA methylation from before to after treatment among 5-year survivors, including an overall loss of methylation at CpG islands and a gain of methylation in non-CpG islands, whereas no significant methylation changes were observed in non-survivors. These treatment-associated methylation changes showed potential prognostic value for long-term survival [13].

Similarly, higher 5-mC and 5-hmC levels were significantly associated with higher tumor grade, where patients expressing higher pretreatment levels of 5-hmC and a trend towards higher 5-mC showed disease progression when treated with neoadjuvant chemotherapy (NACT) [9]. DNA methylation signatures may provide prognostic information in TNBC, with specific differentially methylated regions showing associations with gene expression and overall survival. In one analysis, 23 differentially methylated regions (DMRs) were identified, including 9 DMR-associated genes linked to overall survival, while SPAG6, LINC10606, and TBCD/ZNF750 showed distinct molecular alterations in TNBC compared with controls [10].

These findings collectively suggest that DNA methylation in TNBC should not be viewed as a unidirectional process. Promoter hypermethylation can lead to the silencing of tumor suppressor genes that are crucial for DNA repair, apoptosis, cell-cycle regulation, and differentiation. Conversely, focal or global hypomethylation may result in the activation of oncogenes, retrotransposons, repetitive elements, inflammatory pathways, and genome instability mechanisms. Consequently, the dysregulation of the TNBC methylome represents a context-dependent equilibrium between gene silencing, oncogenic activation, chromosomal instability, treatment adaptation, and biomarker potential. This dual role elucidates why methylation signatures may concurrently serve as diagnostic, prognostic, predictive, and therapeutic-response markers [14].

### 2.2. Histone Modifications

Histone modifications govern the plasticity of TNBC through the coordinated actions of epigenetic writers, erasers, readers, and histone marks. Writers, including histone acetyltransferases (HATs) and Enhancer of Zeste Homolog 2 (EZH2), introduce either activating or repressive modifications. Erasers, such as histone deacetylases (HDACs), remove acetyl groups, thereby altering chromatin accessibility. Readers, exemplified by Bromodomain and extra terminal domain (BET) proteins, identify acetylated histones and maintain enhancer-driven transcriptional programs. Histone marks, such as H3K27me3 and H3K4me3, delineate chromatin states as repressed, active, or bivalent. This writer–eraser–reader framework elucidates the mechanisms by which TNBC cells employ chromatin remodeling to regulate epithelial–mesenchymal transition (EMT), stemness, adaptation to DNA damage, immune evasion, and drug tolerance [5].

Histone lactylation represents an emerging histone modification that links cellular metabolism to epigenetic regulation. It is associated with intracellular lactate levels and can influence chromatin activity and gene transcription. In TNBC, histone lactylation has been implicated in metabolic adaptation and tumor progression, with emerging evidence suggesting a potential role in therapy resistance and the tumor microenvironment.

HATs have long been implicated in TNBC oncogenesis and progression. HATs have been shown to promote EMT—KAT2A-induced EMT in breast cancer cells by activating the TGF-β/Smad pathway. Inhibition of KAT2A reduced the survival, migration, and invasion of MDA-MB-231, an in vitro TNBC model [15].

Another important class of epigenetic regulators implicated in TNBC progression, stemness, and therapy resistance is histone deacetylases (HDACs). HDACs remove acetyl groups from histones, promoting chromatin compaction and transcriptional repression, while also regulating diverse biological processes, including cell proliferation, differentiation, metabolism, apoptosis, and immune regulation. Dysregulated HDAC activity has been implicated in cancer progression by promoting cancer cell survival, suppressing tumor suppressor genes, and altering transcriptional programs associated with treatment adaptation. In TNBC, HDACs have been investigated therapeutically because of their roles in oncogenesis and tumor progression, including regulation of autophagy-related genes such as GABARAPL1 [16]. However, individual HDAC members may exert distinct and context-dependent functions. HDAC7 was found to be downregulated in TNBC samples and associated with survival, while its silencing enhanced TNBC cell proliferation, and its overexpression inhibited proliferation through the NudCD1/GGH axis [17]. In contrast, HDAC8 has been reported to promote TNBC cell migration through regulation of the Hippo-YAP signaling pathway and breast cancer cell dissemination through the AKT/GSK-3β/Snail signaling pathway [18,19].

BET proteins are a family of proteins that regulate gene transcription. Bromodomain-containing protein 4 (BRD4) was found to upregulate the expression of mutant p53, which acts as an oncogene and promotes TNBC progression. Knockdown of BRD4 or treatment with its inhibitor JQ1 suppressed the mutant p53 [20]. HDAC8 has also been reported to promote TNBC cell migration through regulation of the Hippo-YAP signaling pathway and breast cancer cell dissemination through the AKT/GSK-3β/Snail signaling pathway.

Histone modification H3K4me3 has the opposite effect; it acts as an activating mark and opens chromatin at gene promoters, facilitating transcription initiation. The presence of both modifications creates a bivalent domain; in TNBC, this dual activity gives genes the ability to rapidly activate or repress, which contributes to transcriptional plasticity [21]. EZH2 is the catalytic methyltransferase component of Polycomb Repressive Complex 2 (PRC2), which deposits the repressive histone mark H3K27me3. Through this activity, EZH2 contributes to the transcriptional silencing of tumor-suppressive, differentiation-associated, immune-regulatory, and therapy-sensitivity genes. In TNBC, aberrant EZH2 activity can promote proliferation, metastasis, stemness, survival, and drug resistance by maintaining a repressive chromatin environment that favors aggressive cell states [22]. However, EZH2 should not be presented as a uniformly oncogenic enzyme in all contexts, because its function may depend on tumor subtype, interacting signaling pathways, catalytic versus non-catalytic activity, and treatment timing.

Dual inhibition of PI3K–mTOR by paxalisib affects EZH2 by impacting both the classic repressive catalytic p85β-EZH2-H3K27me3 and active EZH2-NF-κB pathways, acting as an upstream blockade. This promotes a favorable mesenchymal-to-epithelial phenotype and inhibits metastasis-initiating cells, cancer stem-cell phenotypes and drug resistance signatures [23]. A promising treatment targeting EZH2 is an AKT-based synergetic therapy. AKT inhibitors synergize with EZH2 inhibitors to promote robust tumor regression in TNBC models and selectively kill TNBC [24].

### 2.3. Chromatin Remodeling and Accessibility

The SWItch/Sucrose Non-Fermentable (SWI/SNF) complex is a multi-subunit chromatin-remodeling complex first identified in yeast that uses ATP to reposition nucleosomes and regulate DNA accessibility. It normally functions to control gene expression by altering chromatin structure, thereby promoting or suppressing the expression of specific genes. In addition, the complex participates in various biological processes and maintains stem-cell pluripotency. Mutations in the SWI/SNF complex occur at high frequencies in various human cancers including TNBC [25]. The SWI/SNF complex acts as a “gatekeeper” of chromatin access for the transcription factors glucocorticoid receptor, GATA6, MYC, and AP-1. Studies have reported that SWI/SNF inhibition shuts down enhancer activity and represses TNBC proliferation, glucocorticoid-induced chemoresistance and invasion in vitro, as well as suppresses tumor growth and metastasis in vivo [26]. Aside from its known function as an E3 ubiquitin ligase, ARID1B forms a novel SWI/SNF complex with SMARCC2 and SMARCB1 that transcriptionally represses the transcription factor and tumor suppressor ZNF382, leading to an increase in TNBC proliferation and migration [27].

Chromatin-accessibility changes are crucial for both EMT and therapy escape in cancer. EMT is a fundamental biological mechanism that causes epithelial cells to acquire a mesenchymal phenotype; therapy escape, on the other hand, involves remodeling chromatin accessibility to silence drug-sensitive programs and activate survival pathways [28,29].

Chemotherapy can select for residual persister cells characterized by altered chromatin states, notably reduced H3K27me3 and increased H3K4me3 at the transcription start sites of genes associated with persister cells. These bivalent or poised chromatin configurations may enable the rapid activation of transcriptional programs related to EMT, stress adaptation, and survival following treatment exposure. The inhibition of EZH2 should be considered independently of chemotherapy, as EZH2 inhibitors directly diminish PRC2-mediated H3K27me3. This action may either suppress tumor-promoting repression or, contingent upon context and timing, facilitate the derepression of persister-associated programs. Consequently, the role of EZH2 in therapy escape should be understood as context-dependent rather than a straightforward linear outcome of chemotherapy [30].

### 2.4. Non-Coding RNAs and Epigenetic Regulation

MicroRNAs play a role in many areas of TNBC development such as proliferation, metastasis, angiogenesis, immune evasion and chemoresistance. The expression or absence of these miRNAs can promote or inhibit cancer development [31]. One such miRNA is miR-34a. miR-34a functions as a tumor-suppressive microRNA and is frequently downregulated across several cancers, including breast cancer. In TNBC models, reduced miR-34a expression has been linked to impaired apoptosis, epithelial–mesenchymal plasticity and treatment resistance [32]. MiR-34a can also be used with other treatments to produce better results. Selinexor is an XPO1 inhibitor that blocks nuclear export of tumor suppressor proteins. When used alone on TNBC cells, it showed anti-proliferative activity, induced apoptosis, and reduced survivin expression and distribution. When used with miR-34a, it produced better anti-proliferative effects, reduced survivin expression, inhibited cell migration, and showed anti-tumor activity effects [33]. Other miRNAs that are of importance in TNBC are the miR-200 family, which has lowered expression in TNBC. They inhibit proliferation, EMT, and migration and induce apoptosis by targeting genes such as CDH1, CASP-3, and TWIST. They also show promise as prognostic and detection markers [31].

Another type of RNA that plays a pivotal role in TNBC is long non-coding RNA (lncRNA). LncRNAs normally function to regulate gene expression at multiple levels; however, in TNBC, their aberrant expression is associated with multiple oncogenic processes, poorer prognosis, and cancer heterogeneity [34]. One such lncRNA is HOX transcript antisense intergenic RNA (HOTAIR). HOTAIR binds to PRC2 and LSD1/CoREST/REST complexes, directing them to specific gene sites, resulting in H3K27 methylation and H3K4 demethylation. HOTAIR can recruit chromatin-modifying complexes and has been associated with tumor progression and drug resistance in breast cancer. Evidence linking HOTAIR to stemness and immune evasion has primarily been derived from non-TNBC models, providing mechanistic support; however, whether HOTAIR exerts similar effects in TNBC remains to be determined [35].

Another lncRNA (similar to HOTAIR) is metastasis-associated lung adenocarcinoma transcript 1 (MALAT1). In contrast to many lncRNAs, MALAT1 is highly conserved, suggesting an important role in gene regulation and development. MALAT1 is upregulated in many cancers and, in TNBC, has been shown to contribute to hypoxia adaptation and promote invasion by sponging miR-129-5p [36]. MALAT1 was shown to be notably overexpressed in TNBC cells compared to other BC subtypes. MALAT1 expression was also correlated with tumor size, lymph node metastasis, and poor prognosis, and inversely correlated with miR-34a and miR-17-5p expression, which were found to be downregulated in patients overexpressing MALAT1 [32]. WTAPP1, a lncRNA, was found to be upregulated in TNBC patients and associated with poor survival. Its expression was inversely correlated with miR-34a expression, with higher levels associated with reduced miR-34a levels and increased expression of EEF2K, a miR-34a target. This dysregulation was associated with increased proliferation, migration, and invasion of TNBC cells [37].

Beyond their roles in post-transcriptional regulation, non-coding RNAs can also participate in broader regulatory networks involving chromatin accessibility and transcription factor binding. In this context, regions with increased chromatin accessibility were enriched for transcription factor motifs, particularly those of CCCTC-binding factor (CTCF) [38].

Another group of RNAs that is implicated in cancer pathogenesis is circular RNAs (circRNAs). They are generated by RNA circularization and mainly function in regulating gene expression [39]. CircRNAs function as miRNA sponges and show promise as possible biomarkers. Several circRNAs were found to be aberrantly expressed in breast cancer cells, including circITCH, which activates Wnt/β-catenin signaling to promote TNBC proliferation, invasion and metastasis by sponging miR-214 and miR-17 [40]. CircPDCD11 promotes TNBC progression by enhancing aerobic glycolysis and correlates with unfavorable survival [41]. CircRNAs are also involved in chemotherapy resistance. In TNBC cells, circUBE2D2 expression was increased and correlated with lymph node metastasis, poor prognosis, and doxorubicin resistance [42]. CircRNAs are also associated with therapy resistance. One mechanism of therapy resistance is through drug efflux. Drug efflux occurs through the ATP-binding cassette (ABC) transporters. These transporters normally function to remove toxic compounds from cells; in cancer cells, they are upregulated and hijacked to remove chemotherapeutic drugs [43].

Chemotherapeutic agents combat cancer cells by inducing DNA damage. Enhanced DNA repair can aid in the development of drug resistance; multiple circRNAs as well as miRNAs are implicated in drug resistance. Flap endonuclease 1 (FEN1) is a DNA replication and repair enzyme associated with drug resistance in chemotherapy. FEN1 was reported to be overexpressed in breast cancer cells, especially after chemotherapeutic treatment. MiR-140 can act as a tumor suppressor by inhibiting FEN1 and is downregulated in breast cancers [44].

Overall, non-coding RNAs provide a flexible regulatory layer that links epigenetic plasticity to TNBC progression and therapy resistance. Tumor-suppressive miRNAs such as miR-34a and the miR-200 family can restrain EMT, survival signaling, and invasion, whereas oncogenic lncRNAs and circRNAs such as HOTAIR, MALAT1, WTAPP1, circITCH, circPDCD11, and circUBE2D2 can promote chromatin repression, miRNA sequestration, glycolytic adaptation, drug efflux, DNA repair, stemness, and metastasis. Rather than functioning as isolated markers, these RNAs form regulatory networks that integrate chromatin state, transcriptional plasticity, tumor microenvironmental signals, and therapeutic stress. This makes ncRNAs important candidates for prognostic modeling, resistance monitoring, and combination-based therapeutic targeting.

### 2.5. 3D Genome Architecture

Even though there are many studies conducted on TNBC, its 3D genome architecture remains poorly elucidated; this has, however, started to change in the past couple of years, with more researchers recognizing the importance of genome reorganization in cancer progression. In TNBC cell lines, topologically associating domains (TADs) were severely perturbed even when compared to other BC cell lines, with only 36% of normal TADs being conserved in BC cells. Furthermore, more than 60% of TADs were weakened in TNBC; these weakened TADs were associated with loss of CTCF occupancy at their boundaries, while strengthened TADs were linked to gain of CTCF occupancy [45]. Changes in genome architecture may also lead to enhancer rewiring. In TNBC, SOX9, an oncogenic transcription factor essential for cancer growth, resides in the most hyper-interacting promoter–enhancer hub. This means that, as a result of genome folding, SOX9 is acted upon by multiple enhancers, leading to increased activity [46]. Genome reorganization may also contribute to transcriptional reprogramming under therapeutic stress and therapy resistance.

The characterization of changes in 3D genome organization during TNBC progression from a primary (PR) drug-sensitive state to a carboplatin-resistant (CR) state revealed significant alterations in genome architecture. CR tumors showed an increased number of short-range chromatin interactions and strengthened TADs. CR tumors also showed a 150% increase in chromatin loops; these loops were longer than those present in PR tumors, suggesting increased long-distance interactions [47]. Another study linking doxorubicin resistance to 3D chromatin reorganization showcased that doxorubicin-resistant BC cells had an increased number of chromatin loops that were shorter.

### 2.6. Epigenetic Drivers of Phenotypic Plasticity

Many factors play a role in the phenotypic plasticity and heterogeneity of TNBC; one of the prominent contributors is EMT and its reverse, known as MET. To maximize tumor progression, cancer cells employ dynamic switching between EMT and MET states. EMT promotes escape and invasion; after invasion, the cells switch to MET to promote colonization and growth [48]. This dynamic switching of states is called epithelial–mesenchymal plasticity (EMP). Many factors regulate EMP; one such factor is the tumor microenvironment (TME). Microenvironmental cues from the TME regulate EMP. Some EMT inducers present in the TME include EGFs, FGFs and interleukins such as IL-6 and IL-8, with the most potent EMT inducer being TGF-β. BMP-7 and BMP-5 (bone morphogenic protein-5/7) have been shown to inhibit EMT with BMP-5 targeting TGF-induced EMT [49,50]. EMP plays a major role in therapy resistance. In chemotherapy, cancer cells displaying resistance to drugs such as cisplatin, paclitaxel and TRAIL were found to display EMT and mesenchymal features [51].

**Figure 1:**
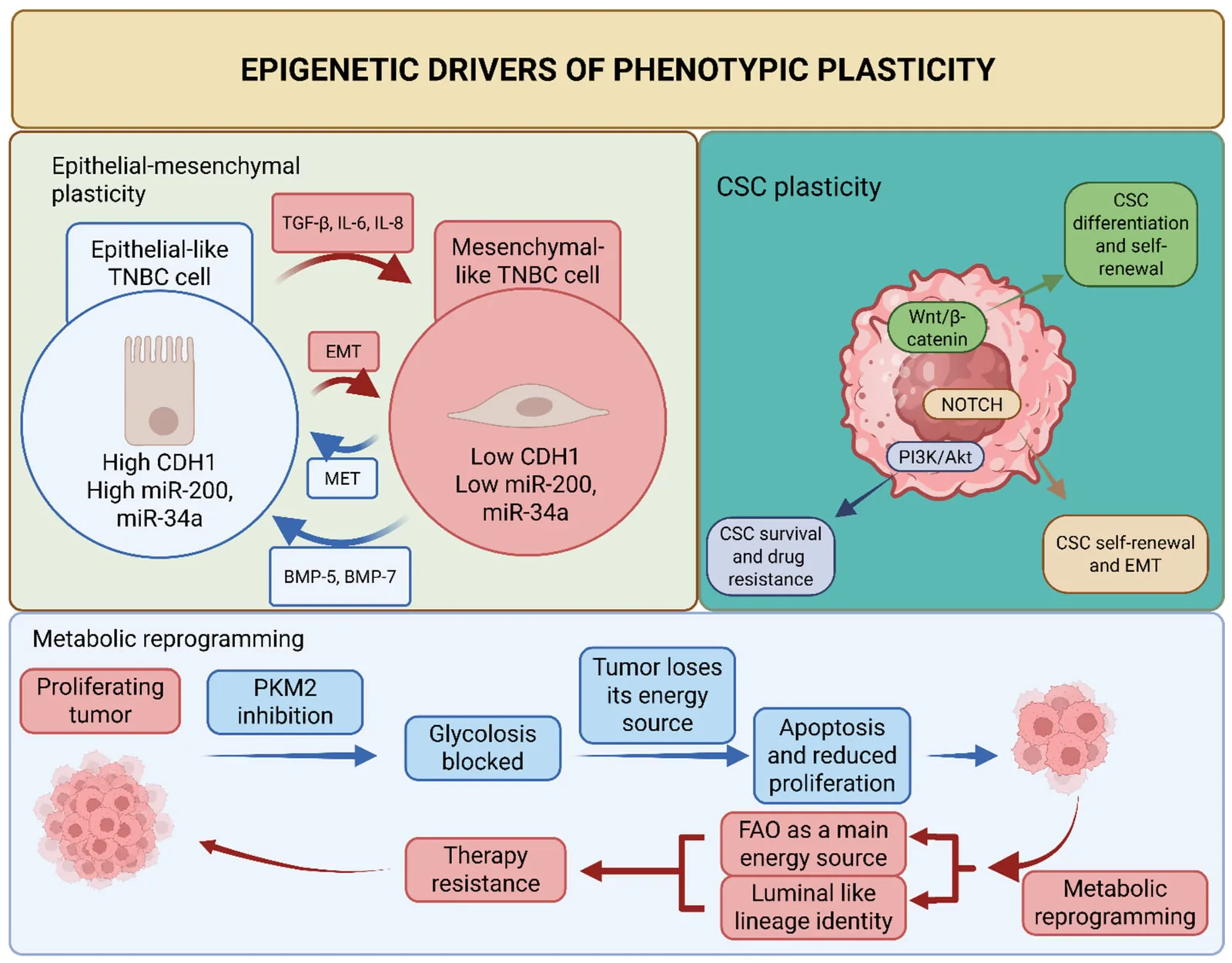
Epigenetic drivers of phenotypic plasticity in TNBC. Epigenetic mechanisms regulate three major adaptive processes that promote tumor progression and therapeutic resistance. They orchestrate epithelial–mesenchymal plasticity through EMT/MET transitions, alter cancer stem-cell (CSC) properties by activating key signaling pathways including Wnt/β-catenin, NOTCH, and PI3K/AKT, and drive metabolic reprogramming to support survival under therapeutic stress. These interconnected programs enable dynamic changes in cellular identity, enhance stemness, facilitate metastasis, and promote drug resistance. Red elements indicate therapy resistance-related processes, whereas blue elements indicate apoptosis-related or tumor-suppressive processes. Together, they illustrate how epigenetic regulation integrates phenotypic plasticity with metabolic adaptation to sustain aggressive TNBC behavior.

Breast cancer stem cells (BCSCs) are a subpopulation of cancer cells with stem-cell-like properties such as self-renewal. They control many facets of tumor progression, including chemoresistance, metastasis, tumorigenesis, and relapse [52]. Just like non-stem-cell cancer cells, BCSCs are affected by epigenetic regulation; dysregulation of the epigenome, especially at genes related to growth and pluripotency, has been linked to stemness [53]. Epigenetic regulation also contributes to the maintenance of stem-like states in TNBC. DNMT1-dependent regulatory networks can reinforce stemness-associated transcriptional programs and support tumorigenic properties, illustrating a direct link between DNA methylation machinery and CSC plasticity [54]. Cancer stem-cell plasticity is one of the factors that makes CSCs remarkably difficult to treat. In TNBC, CSC plasticity is regulated by signals from various signaling pathways and the TME. Key signaling pathways promoting CSC plasticity include Notch, which drives CSC self-renewal and EMT; Wnt/β-catenin, which promotes CSC differentiation and self-renewal; and PI3K/Akt, which contributes to CSC persistence by enhancing CSC survival and drug resistance [55]. Cancer cell lineage plasticity can be defined as the ability of cells to change their identity or lineage, resulting in them expressing a new phenotype; it is often triggered in TNBC when the cell is under different stresses such as therapy-induced stress. Lineage plasticity has been shown to cause resistance, metastasis, therapy escape, adaptation and survival of tumor cells [56], as shown in Figure 1.

Taken together, these mechanisms define the molecular machinery that permits state switching, but their presence alone does not establish treatment resistance. Section 3 therefore evaluates the contexts in which these regulators have been linked to drug-tolerant residual states, repair restoration, immune escape, metabolic adaptation or microenvironment-mediated protection, while distinguishing direct TNBC evidence from broader mechanistic inference.

## 3. Adaptive States and Therapy Resistance

Epigenetic plasticity drives treatment resistance in triple-negative breast cancer by enabling reversible changes in chromatin, DNA methylation, and non-coding RNAs that shift cells into stem-like and drug-tolerant states. Therapy can further reprogram tumors, producing persister cells, EMT activation, and metabolic adaptation. These processes promote survival without new mutations, leading to relapse, metastasis, and durable resistance, while revealing targets for epigenetic combination therapies [5,57].

### 3.1. Chemotherapy Resistance

Epigenetic plasticity plays a crucial role in the development of chemotherapy resistance in TNBC, facilitating adaptive modifications that enhance drug efflux and the avoidance of apoptosis. Within TNBC cells, abnormal DNA methylation patterns and histone modifications lead to the upregulation of efflux pumps, including ABC transporters, thereby diminishing intracellular drug concentrations during treatment with agents such as doxorubicin and taxanes. Furthermore, these epigenetic changes contribute to the silencing of pro-apoptotic genes, such as BIM and PUMA, which enable cancer cells to withstand the cytotoxic stress caused by cisplatin [16,58].

Promoter methylation of DNA-repair genes should be interpreted as a dynamic rather than uniformly resistance-promoting event. BRCA1 promoter methylation can suppress BRCA1 expression and establish homologous-recombination deficiency. Under platinum exposure, however, loss of BRCA1 methylation can restore BRCA1 expression and promote acquired platinum resistance, demonstrating the adaptive nature of epigenetic DNA-repair regulation in TNBC [16,59].

Doxorubicin resistance in TNBC is often associated with epigenetic activation of drug-efflux programs, including ABC transporters such as MDR1/ABCB1, which encodes P-glycoprotein. MDR1 upregulation should be linked to chromatin states that enhance transcriptional accessibility, including promoter or enhancer activation, but not reduced acetylation unless supported by the study. HDAC inhibitors may restore chemosensitivity by reprogramming chromatin accessibility, altering apoptosis-related gene expression, and disrupting survival programs, but effects are context-dependent [60,61].

**Figure 2:**
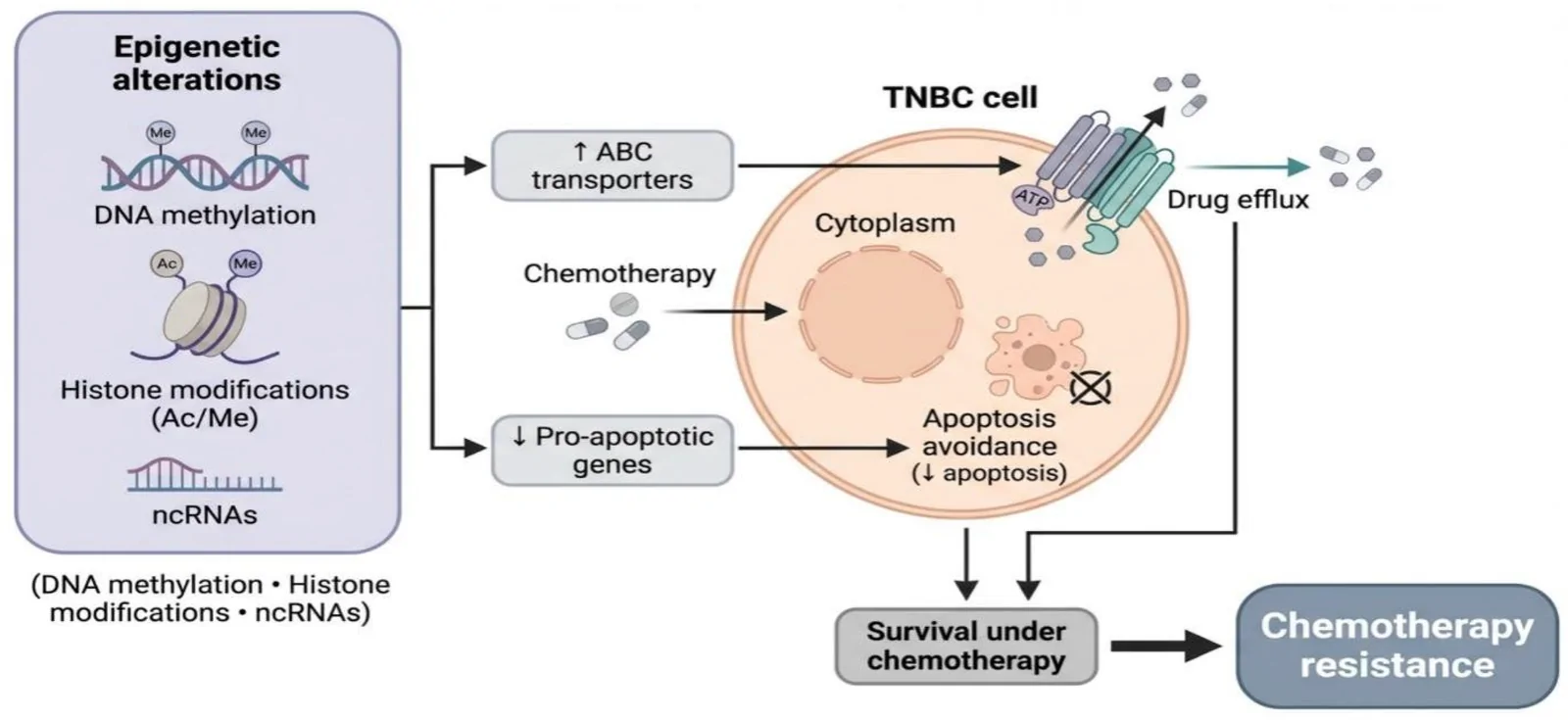
Epigenetic mechanisms underlying chemotherapy resistance in TNBC. Epigenetic alterations, including DNA methylation, histone modifications, and non-coding RNAs (ncRNAs), reprogram gene expression to promote chemoresistance. These changes increase ATP-binding cassette (ABC) transporter expression, enhancing drug efflux, while simultaneously suppressing pro-apoptotic genes to reduce chemotherapy-induced cell death. Together, these mechanisms enable TNBC cells to survive cytotoxic treatment despite intracellular drug exposure. The coordinated epigenetic regulation of drug transport and apoptosis ultimately drives persistent chemotherapy resistance and tumor recurrence.

Hypomethylation of LINE-1 elements has been associated with a diminished response to taxane–doxorubicin treatments, which reflects the genomic instability that contributes to resistance across various chemotherapy regimens. Integrated epigenomic profiling indicates that gains in H3K4me3 at stemness genes, such as SOX2, are predictive of taxane unresponsiveness, thereby highlighting the significance of plasticity. These epigenetic changes, working together, promote changes in cellular phenotypes, leading to either a dormant or a mesenchymal state [62]. As illustrated in Figure 2, epigenetic alterations, including DNA methylation, histone modifications, and ncRNA regulation, drive chemoresistance in TNBC by enhancing drug efflux and suppressing apoptosis.

### 3.2. Resistance to Targeted Agents

Epigenetic plasticity plays a crucial role in the development of resistance to PARP inhibitors in TNBC, particularly in BRCA1/2 wild-type tumors lacking germline alterations that confer synthetic lethality. In some cancers, reversible epigenetic changes can restore HR, allowing cells to repair DNA damage caused by PARP inhibitors. For instance, hypermethylation of the BRCA1 promoter results in gene silencing. This induces a transient state of BRCA insufficiency, rendering cells susceptible to olaparib or talazoparib. The subsequent reactivation of BRCA1 by demethylation may diminish the efficacy of these medications [63,64].

In this context, dynamic changes in DNA methylation can alter BRCA1 expression and homologous-recombination capacity, thereby influencing sensitivity to PARP inhibition. Specifically, reduced TET2 activity stabilizes hypermethylated states before resistance develops through compensatory hypomethylation. Research shows that EZH2-driven H3K27me3 at HR genes, such as RAD51, initially prevents repair. However, when EZH2 is lost or inhibited, this repression is lifted, restoring fork stability and HR activity in BRCA wild-type TNBC cells. This adaptability allows for phenotypic changes without requiring genetic mutations [65].

Crosstalk between epigenetic modifications and DNA damage-repair (DDR) pathways exacerbates resistance to PARP inhibitors. Deficiencies in histone acetylation, which are mediated by HDACs, hinder the recruitment of 53BP1 to double-strand breaks, thereby promoting HR over non-homologous end joining and circumventing PARPi trapping. In TNBC models that are proficient in BRCA1/2, hyperacetylation of chromatin by HDAC6 stabilizes replication forks, thus preventing their collapse and the consequent synthetic lethality [63,66]. PARG loss has been associated with reduced PARP trapping and altered DNA-repair dependence in broader tumor models. This mechanism provides a plausible route to PARP-inhibitor resistance, but its contribution to TNBC requires direct validation [67].

Chromatin remodeling and histone modifications contribute to the development of resistance to PARP inhibitors in TNBC. The loss of ARID1A and alterations in SWI/SNF complexes are associated with increased H3K27ac levels, whereas BET proteins maintain acetylation. Furthermore, pre-existing heterogeneity in H3K9me2/3 and enhancer reprogramming at MYC promote the expression of efflux pumps and anti-apoptotic factors. In preclinical models, targeting BET or EZH2 has been shown to restore sensitivity to PARP inhibitors [25,68].

### 3.3. Immune Evasion and Checkpoint-Blockade Resistance

Epigenetic remodeling promotes immune escape and checkpoint-blockade resistance in TNBC by suppressing antigen presentation, weakening interferon-responsive transcription, altering PD-L1 inducibility, repressing STING-associated viral-mimicry pathways, and reshaping macrophage and T-cell states within the tumor microenvironment. Hypermethylation of antigen-presentation genes, repressive histone modifications at immune-response loci, and chromatin-mediated silencing of JAK/STAT and interferon signaling can reduce cytotoxic T-cell recognition even when PD-1/PD-L1 inhibitors are administered. Therefore, immune resistance in TNBC should be interpreted as an ecosystem-level process involving tumor-cell chromatin states, antigen-processing machinery, interferon signaling, immune-checkpoint regulation, and stromal or macrophage-mediated immunosuppression [62,69,70].

Furthermore, this suppression affects pathways linked to the interferon response. Epigenetic silencing of elements within the JAK/STAT signaling pathway diminishes the expression of type I IFN-inducible genes, including IFIT1 and MX1. Moreover, DNMT1-mediated methylation of IFNAR1 promoters impedes STAT1 phosphorylation, thereby attenuating anti-viral and immune-stimulatory responses that are augmented by checkpoint inhibitors. Studies using ChIP-seq data show that HDAC1 reinforces these repressive marks, which predicts poor responses to anti-PD-1 therapy in PD-L1-positive TNBC patient groups [71,72].

In BC cells, TET2 was reported to suppress PD-L1 transcription by recruiting HDAC1/2 to the PD-L1 promoter and reducing histone H3 acetylation; TET2 loss increased PD-L1 expression. Because this study was not limited to TNBC or to immunotherapy-treated cohorts, its relevance to TNBC checkpoint resistance should be considered indirect [73,74].

Epigenetic disruption of antigen-processing and MHC-I pathways has been implicated in immune escape across cancer models. Such findings provide mechanistic plausibility for impaired tumor recognition in TNBC, but their direct contribution to checkpoint-blockade resistance in TNBC remains incompletely validated [75].

Wide-ranging research on cancer shows that chromatin control mechanisms have the potential to inhibit STING–interferon signal transmission and antigen-presenting processes, as well as immune control signaling mechanisms. Despite this, there is a lack of sufficient evidence for the existence of certain elements of this control network in TNBC [76].

### 3.4. Metabolic Reprogramming

Epigenetic plasticity significantly impacts metabolic reprogramming in TNBC, especially through the modulation of glycolysis and fatty-acid metabolism, thereby facilitating the adaptation of tumor cells to environments with limited nutrients. The expression of glycolytic enzymes, including hexokinase 2 (HK2) and pyruvate kinase M2 (PKM2), is dynamically regulated by histone modifications and DNA methylation. These enzymes are upregulated through EGFR and HIF-1α signaling, which promotes the Warburg effect and lactate production, thus supporting accelerated proliferation [77,78].

When PKM2-dependent glycolysis was inhibited, TNBC models shifted toward fatty-acid oxidation and a luminal-like transcriptional state, creating a compensatory metabolic dependency. This finding links metabolic switching to lineage plasticity, but its therapeutic relevance remains preclinical [65].

At the same time, epigenetic changes enhance the uptake of fatty acids through transport proteins like CD36 and lipoprotein lipase (LPL). These changes also affect acetyl-CoA carboxylase (ACC), which helps balance the creation of new lipids and β-oxidation. This process provides lipids for building membranes and storing energy [79,80].

This metabolic shift is associated with therapeutic resistance; specifically, epigenetically controlled glycolytic adaptations bolster cancer stem-like cells, enabling them to evade apoptosis instigated by chemotherapeutic agents. For example, augmented fatty-acid oxidation provides survival benefits amidst oxidative stress caused by DNA-damaging compounds, thereby connecting lipid metabolism to platinum resistance in TNBC models. Evidence from broader cancer models suggests that histone lactylation can connect increased lactate production with immunosuppressive transcriptional programs. Its specific contribution to immune dysfunction and immunotherapy resistance in TNBC remains to be established [81].

Epigenetic regulation of glutamine- and serine-metabolism genes can support nucleotide synthesis, redox homeostasis and maintenance of stem-like states under nutrient or treatment stress [82,83,84,85]. The relative contribution of each metabolic pathway remains context-dependent and requires functional validation in TNBC models.

### 3.5. Microenvironment-Driven Epigenetic Resistance

In the tumor microenvironment of TNBC, hypoxia primarily drives epigenetic resistance through chromatin remodeling controlled by hypoxia-inducible factor 1α (HIF1α). HIF1α recruits HDAC1 and PRC2 to influence gene promoters like IFNG and TNF. This process leads to the addition of repressive histone marks, such as H3K27me3, without causing widespread epigenetic changes. Consequently, TNBC cells become trapped in a state that avoids the immune system and is resistant to treatment. This is mainly due to a decrease in CD8+ T-cell infiltration and the production of IFNγ [86].

Evidence from broader solid-tumor models indicates that cancer-associated fibroblasts (CAFs) can undergo epigenetic reprogramming involving DNA methylation and TET-dependent mechanisms, thereby supporting immunosuppressive stromal states. These findings provide mechanistic plausibility for TNBC but have not been fully validated as TNBC-specific resistance mechanisms [87].

Epigenetic regulation of tumor-associated macrophage states through EZH2- and HDAC-associated programs has been described across tumor models and may contribute to immunosuppressive polarization. However, direct causal evidence establishing this mechanism as a driver of therapeutic resistance in TNBC remains limited [70].

Chronic hypoxia in TNBC tumors, in addition to its direct gene repression, triggers genome-wide changes in DNA methylation. This is mediated by the stabilization of TET enzymes via HIF1α, which, paradoxically, facilitates demethylation at pro-metastatic loci while concurrently silencing apoptosis pathways. This specific epigenetic modification not only improves cell survival in low-oxygen environments but also interacts with metabolic reprogramming. Specifically, lactate accumulation further amplifies HDAC activity, thereby reinforcing resistance to chemotherapeutic agents like doxorubicin. As a result, hypoxic environments develop into self-sustaining centers of plasticity, thereby complicating the targeting of therapeutic interventions [86,88].

CAFs, including TGF-β, induce HIF1α in neighboring immune cells, which subsequently spreads repressive H3K9me3 marks across antigen-presentation genes. This feedback loop arises from the interaction between hypoxic signaling and the dynamics of the stroma and immune system. This coordinated remodeling fosters an exhausted T-cell phenotype, distinguished by the epigenetic silencing of cytotoxic effectors and the persistent expression of PD-1. EZH2 inhibitors have shown promise in preclinical models by disrupting this axis, thus reinstating anti-tumor immunity and preventing TNBC relapse [70,89,90]. Table 1 provides an integrated overview of the major epigenetic mechanisms underlying therapeutic resistance in TNBC, together with representative biomarkers and candidate molecular targets. Because the evidence supporting epigenetic resistance mechanisms varies substantially in experimental source and translational maturity, Table 1 distinguishes TNBC-specific findings from evidence extrapolated from broader breast cancer or other cancer models.

As summarized in Table 1, most proposed epigenetic resistance mechanisms remain supported by cell-line, organoid or animal studies. The strongest translational evidence concerns dynamic BRCA1 promoter methylation and treatment-associated chromatin plasticity; however, neither has been prospectively validated as a routine biomarker for treatment selection. Mechanisms derived from other breast cancer subtypes or malignancies should be regarded as indirect evidence until independently reproduced in TNBC, preferably using paired pretreatment and post-progression specimens.

## 4. Resistance-Focused Biomarkers

Epigenetic biomarkers are gaining prominence in the study of TNBC, a subtype of the disease that lacks established therapeutic strategies. These biomarkers, which detect reversible molecular alterations such as DNA methylation and chromatin remodeling, present novel avenues for treatment selection and prognostic predictions. This is particularly significant considering the aggressive behavior and heterogeneous clinical presentations characteristic of this specific cancer [93].

### 4.1. Biomarkers of Epigenetic Adaptation and Treatment Resistance

Tissue-based methylation profiles can provide a baseline view of epigenetic state in TNBC. DNA methylation patterns at CpG islands of tumor suppressor genes can distinguish malignant from nonmalignant breast tissue, while genome-wide signatures can stratify TNBC into biologically distinct groups with different clinicopathological features [94,95]. In the context of this review, these patterns are considered primarily as molecular-stratification and longitudinal-comparison tools rather than as established population-screening tests.

Promoter methylation involving genes such as BRCA1, CDH1, PTEN, and RASSF1 has been reported in TNBC, but the clinical utility of individual loci varies across cohorts and assays. Composite methylation signatures may therefore be more informative for molecular classification and resistance-oriented longitudinal studies than single-gene markers [91,92].

Liquid-biopsy approaches provide a minimally invasive route for longitudinal epigenetic monitoring. Circulating tumor DNA (ctDNA) can carry tumor-associated methylation patterns that reflect the primary lesion, and plasma cell-free DNA studies have identified differentially methylated regions capable of distinguishing TNBC from control samples [10,96]. For the present review, the most relevant application is serial assessment of treatment response, residual disease, and emerging resistance; early-detection applications require independent prospective validation.

Circulating microRNAs, long non-coding RNAs, and methylation changes detected in circulating tumor cells may likewise capture evolving tumor states. Their principal value for resistance research is the feasibility of repeat sampling over time, which can support longitudinal assessment of treatment response and resistance evolution without repeated tissue biopsy [84,85].

### 4.2. Predictive Biomarkers for Therapy Response

Epigenetic biomarkers are increasingly recognized as valuable predictors of therapeutic efficacy in TNBC, a subtype that generally exhibits resistance to conventional hormone and HER2-targeted therapies. DNA methylation patterns are critical in influencing chemotherapy sensitivity through the modulation of genes implicated in apoptosis, DNA-repair processes, and drug metabolism pathways. Therefore, specific methylation signatures, ascertained before the commencement of treatment, can distinguish between patients likely to exhibit a positive response and those unlikely to benefit from neoadjuvant chemotherapy, thus supporting the formulation of personalized treatment approaches. The prognostic significance of DNA methylation in TNBC is strongly context-dependent. Distinct methylation-defined tumor states are associated with different transcriptional programs, clinicopathological characteristics, and clinical outcomes, indicating that prognostic interpretation should consider the broader methylation landscape rather than individual loci in isolation [82,83].

Pretreatment DNA methylation profiling has identified specific methylation signatures associated with response to neoadjuvant chemotherapy in TNBC. Combinations of differentially methylated loci can distinguish treatment-responsive from non-responsive tumors, supporting their potential use as predictive biomarkers, although prospective clinical validation remains necessary [82]. Epigenetic modifications can also influence the efficacy of immunotherapeutic interventions. Specifically, the silencing of genes responsible for antigen presentation or those implicated in interferon signaling via methylation can impede the immune system’s ability to identify tumors. This, in turn, may diminish the effectiveness of immune-checkpoint inhibitors. Conversely, reduced methylation of immune-related genes could enhance the immunogenicity of the tumor. Therefore, analyzing a patient’s epigenetic profile could help identify those who are most likely to benefit from immunotherapy, which is very important for treating TNBC [97,98].

Histone-based biomarkers offer additional predictive capabilities. Changes in histone acetylation and methylation influence chromatin accessibility and the transcriptional programs linked to drug resistance. Likewise, non-coding RNAs, including miR-21, miR-34a, MALAT1, and HOTAIR, modulate pathways associated with proliferation and metastasis; their expression levels are correlated with therapeutic results. Consequently, the integration of histone marks and ncRNA profiles could enhance predictive accuracy beyond the scope of DNA methylation alone [97,99].

### 4.3. Prognostic Biomarkers

Prognostic epigenetic biomarkers provide significant information regarding survival forecasts, the probability of disease recurrence, and the progression of TNBC. Increased methylation within tumor suppressor genes often correlates with a more aggressive disease presentation and less favorable prognoses [91,100].

Conversely, some methylation profiles have been associated with improved outcomes, underscoring the context-dependent nature of epigenetic regulation. In selected populations, methylation changes in immune or inflammatory genes have been linked to survival, indicating that the direction of prognostic association depends on the pathway, cellular compartment, and patient cohort. This variability supports the use of multigene signatures rather than isolated loci [101,102].

Large-scale analyses have identified crucial “hub” genes within the context of TNBC. The expression profiles of these genes, along with their associated DNA methylation patterns, correlate directly with patient survival. Specifically, the demethylation of certain cell-cycle genes, including KIF11, CCNB1, and PLK1, frequently results in their overexpression. This overexpression, in turn, accelerates tumor-cell proliferation and is associated with poorer clinical prognoses. Consequently, this finding highlights the role of epigenetic modifications in fostering aggressive tumor behaviors and increasing the probability of metastasis [103].

Because epigenetic states can change during treatment, longitudinal methylation, chromatin, or non-coding-RNA measurements may provide dynamic information that static genomic markers cannot capture. Serial liquid-biopsy analysis is therefore being investigated for monitoring residual disease, emerging resistance, and recurrence, although its clinical utility in TNBC has not yet been established [104,105].

### 4.4. Single-Cell Epigenomics and Heterogeneity

TNBC is characterized by substantial intratumoral heterogeneity, which contributes to therapeutic resistance and recurrence. Bulk analyses can obscure rare but clinically relevant subpopulations. Single-cell chromatin-accessibility approaches, including scATAC-seq, can resolve cell-state-specific regulatory programs; however, direct TNBC chromatin-level evidence remains less mature than the single-cell transcriptomic literature [106,107].

When chromatin-accessibility profiling is integrated with single-cell RNA sequencing, regulatory state can be linked to gene-expression programs. This multimodal approach can help identify stem-like, immune-evasive, or treatment-persistent populations and generate candidate mechanisms for resistance and relapse [106,107].

Single-cell transcriptomic profiling has revealed substantial cellular heterogeneity in TNBC and identified treatment-associated immune and tumor-cell states linked to therapeutic response [108,109]. Where chromatin-accessibility measurements are available, integration with transcriptomic data can further resolve regulatory programs associated with resistant phenotypes [106,107].

Single-cell and spatial profiling can also clarify interactions among malignant, immune, and stromal compartments that influence treatment response. These ecosystem-level maps are useful for generating resistance hypotheses and identifying candidate cellular interactions for combination strategies, but transcriptomic or spatial-expression measurements should not be interpreted as direct epigenomic evidence unless chromatin or methylation is measured [106,109,110,111]. Table 2 summarizes these biomarker frameworks and their principal implementation barriers.

Spatial profiling can localize resistant or immunosuppressive cellular niches within tissue architecture, providing complementary information to single-cell analyses [110,111,112].

These approaches can identify rare cellular populations associated with relapse or treatment non-response, but spatial transcriptomic findings should not be treated as direct epigenomic measurements unless chromatin or methylation is assayed [110,111,112].

Longitudinal single-cell sampling, when available, can track changes in cellular states during therapy and generate hypotheses about adaptive resistance [108,109].

Taken together, single-cell chromatin, transcriptomic, and spatial profiling provide complementary rather than interchangeable views of TNBC heterogeneity. Direct longitudinal epigenomic validation remains limited, so these technologies are best viewed as hypothesis-generating platforms for resistance-biomarker discovery [106,111,112].

**Table 2:** Advanced epigenetic biomarker domains in TNBC, their clinical relevance, and implementation challenges, with evidence from broader breast cancer or non-TNBC models interpreted as indirect rather than TNBC-specific validation.

Biomarker Category	Representative Biomarkers or Approaches	Potential Clinical Relevance in TNBC	Citations
General epigenetic biomarkers	DNA methylation profiles, chromatin-remodeling alterations, histone modifications and non-coding-RNA expression	May support molecular stratification, outcome estimation and treatment-response assessment in heterogeneous TNBC populations	[93]
Tissue-based methylation biomarkers	CpG-island methylation involving tumor suppressor genes such as BRCA1, CDH1, PTEN and RASSF1	May support baseline molecular stratification and comparison of treatment-naive versus treatment-exposed states; not established as a population-screening test	[91,92,94,95]
Circulating epigenetic biomarkers	Methylated circulating tumor DNA, circulating miRNAs and lncRNAs, and methylation changes detected in circulating tumor cells	Under investigation for longitudinal assessment of treatment response, residual disease, and resistance evolution	[10,84,85,96]
Chemotherapy-response biomarkers	Methylation signatures involving apoptosis, DNA-repair and drug-metabolism pathways	May help identify patients more or less likely to respond to neoadjuvant chemotherapy, although prospective clinical validation remains limited	[82,83,113]
Immunotherapy-response biomarkers	Epigenetic alterations affecting antigen-presentation and interferon-response pathways	May complement established immune biomarkers when evaluating response or resistance to immune-checkpoint inhibitors	[97,98]
Multimodal predictive biomarkers	Histone-acetylation or methylation patterns and non-coding RNAs, including miR-21, miR-34a, MALAT1 and HOTAIR	Associations with treatment response and resistance have been reported, primarily in retrospective cohorts and preclinical models	[97,99]
Prognostic DNA methylation biomarkers	Promoter hypermethylation involving BRCA1, PTEN, GSTP1 and other tumor-suppressive genes	Certain alterations have been associated with aggressive clinicopathological features or survival outcomes, but findings are not consistent across cohorts	[91,100]
Prognostic pathway signatures	Methylation profiles involving immune and inflammatory pathways and altered methylation of proliferation-associated genes such as KIF11, CCNB1 and PLK1	May define prognostically distinct biological states; the direction of the association depends on the gene, cellular compartment and patient population	[101,102,103]
Longitudinal circulating biomarkers	Treatment-associated changes in circulating DNA methylation, chromatin-derived signals or non-coding-RNA abundance	Potentially useful for monitoring residual disease, emerging resistance and recurrence, but clinical utility has not been established	[104,105]
Single-cell chromatin profiling	Single-cell assay for transposase-accessible chromatin sequencing, alone or integrated with single-cell RNA sequencing	Resolves cell-specific chromatin accessibility and may identify stem-like, immune-evasive or treatment-persistent cellular populations	[106,107]
Spatial and longitudinal profiling	Cell-resolved spatial and single-cell maps across malignant, immune, and stromal compartments, with chromatin-level interpretation restricted to assays that directly measure epigenetic state	Can generate mechanistic hypotheses concerning resistance-associated cellular interactions and candidate combination strategies	[106,110,111,112]

## 5. Therapeutic Vulnerabilities and Clinical Evidence

The translational maturity of epigenetic targets in TNBC is uneven. DNMT and HDAC inhibitors have entered breast cancer trials and provide measurable pharmacodynamic and immune effects, but clinical benefit remains inconsistent, and toxicity may limit combination dosing. BET, EZH2 and demethylase inhibitors have compelling context-specific preclinical rationales, yet predictive biomarkers and TNBC-specific clinical validation remain limited. Accordingly, this section separates clinical signals from preclinical vulnerability and hypothesis-generating mechanisms rather than treating all epigenetic targets as equally drug-ready [114].

Epigenetic plasticity is relevant to TNBC therapy because reversible chromatin states can support survival under hypoxia, immune pressure, and drug exposure [57,92]. By enabling transitions among epithelial, mesenchymal, stem-like, and other stress-adapted states, these mechanisms can contribute to heterogeneous treatment responses. Therapeutic targeting of epigenetic regulators is therefore most plausibly considered as a strategy for re-sensitization or priming in selected contexts, rather than as a universal solution for TNBC [5].

### 5.1. Epigenetic Drug Therapies in TNBC

Epigenetic therapy is being investigated as a strategy to modulate treatment-associated plasticity in TNBC. Its rationale is strongest when a defined epigenetic dependency can be linked to a resistant state and paired with a biomarker or rational combination partner [92].

Epigenetic agents investigated in TNBC act at distinct regulatory levels. DNMT inhibitors such as decitabine and azacitidine reduce maintenance methylation and may reactivate silenced immune or tumor-suppressive programs, whereas HDAC inhibitors increase protein and histone acetylation but have broad, context-dependent effects and dose-limiting toxicities. BET inhibitors suppress enhancer-dependent transcriptional programs, while EZH2 inhibitors reduce PRC2-mediated H3K27me3; these classes should not be grouped mechanistically because they target chromatin reading and writing, respectively. LSD1 and other KDM inhibitors remain predominantly preclinical in TNBC and may affect stem-like or immune-evasive states. Across classes, monotherapy activity in solid tumors has generally been limited, supporting biomarker-guided combinations rather than unselected use [6,115]. Table 3 provides an overview of major epigenetic drug classes investigated in TNBC, highlighting their mechanisms of action, therapeutic relevance, and implementation challenges.

### 5.2. Preclinical Evidence

Preclinical studies in TNBC have consistently demonstrated that epigenetic therapies can work in both intrinsic and acquired resistance to conventional chemotherapy. DNA methyltransferase inhibitors (DNMTis), especially decitabine, have shown the ability to reactivate silenced tumor suppressor genes, induce apoptosis, and inhibit tumor-cell proliferation in TNBC models [116]. These effects are particularly pronounced in contexts where chemotherapy is ineffective, where DNMT expression levels correlate with treatment efficacy, and where decitabine facilitates the degradation of DNMT proteins in both in vitro systems and patient-derived xenograft (PDX) models. Histone deacetylase inhibitors (HDACis), particularly in conjunction with DNMT inhibitors (DNMTis), enhance cancer therapy by modifying the transcriptional networks that regulate survival, metastasis, and drug resistance [16]. This dual epigenetic targeting has been effective in reversing EMT, reducing cell proliferation, preventing invasion and migration, and triggering apoptosis in aggressive TNBC cell lines. These alterations indicate a broader epigenetic reprogramming that diminishes the aggressiveness of cancer cells [5].

In vivo xenograft and patient-derived models support the possibility that epigenetic therapy can reduce tumor growth or enhance sensitivity to other treatments in selected TNBC contexts. These preclinical results are useful for defining mechanisms and treatment sequences, but they should not be interpreted as established clinical efficacy. Their translational value depends on model context, dose, scheduling, and biomarker-defined patient selection [116,117].

### 5.3. Clinical Trial Landscape

Clinical evidence for epigenetic therapy in TNBC remains limited and currently supports biological activity and combination feasibility more strongly than established therapeutic efficacy. In the randomized ENCORE 602 trial (NCT02708680), adding the HDAC inhibitor entinostat to atezolizumab in previously treated advanced TNBC did not significantly improve progression-free survival; median PFS was 1.68 versus 1.51 months and objective response rates were 10.0% versus 2.4% [118]. In a phase II neoadjuvant window study (NCT02957968), sequential decitabine and pembrolizumab increased stromal lymphocyte infiltration and altered immune-related biomarkers in HER2-negative breast cancer, but the cohort included both TNBC and hormone-receptor-positive disease and the independent contribution of decitabine to pathological complete response could not be isolated [119]. A phase I study (NCT05673200) is currently evaluating oral decitabine/cedazuridine (ASTX727) with paclitaxel and pembrolizumab in metastatic or unresectable TNBC; the registry currently lists the study as temporarily closed to accrual, and efficacy has not been established [120]. Collectively, these studies support continued investigation of biomarker-guided epigenetic combinations but do not justify routine use of epigenetic agents for TNBC outside clinical trials (see Table 4).

### 5.4. Rational Combination Strategies

Due to the plasticity of TNBC, the durability of epigenetic monotherapy is limited. Therefore, combination therapies must address both tumor populations and therapy-induced adaptive states. These combinations should align with specific objectives, such as epigenetic priming prior to chemotherapy, restoration of DNA-damage susceptibility before PARP inhibition, immune reactivation preceding checkpoint blockade, or suppression of microenvironment-driven chromatin adaptation. The success of these strategies is contingent upon the sequence of administration, dose intensity, toxicity management, pharmacodynamic monitoring, and the use of predictive biomarkers [121].

#### 5.4.1. Combination with Immunotherapy

Epigenetic therapy may enhance immunotherapy by reactivating antigen-presentation and interferon-related programs. However, the clinical evidence is mixed: immune-modulating pharmacodynamic effects have been observed, whereas ENCORE 602 did not show a significant progression-free-survival benefit from adding entinostat to atezolizumab. Accordingly, epigenetic–immunotherapy combinations remain investigational and should be evaluated using biomarker-guided and sequence-aware designs [118,122].

#### 5.4.2. Combination with Chemotherapy

Integrating epigenetic therapy with chemotherapy is a logical approach, as cytotoxic agents can induce drug-tolerant states characterized by chromatin remodeling. Agents such as DNMTi, HDACi, BETi, or EZH2i have the potential to enhance therapeutic outcomes by reactivating apoptotic genes, diminishing persister cell programs, compromising DNA-damage tolerance, and restricting transcriptional escape. The sequence of administration is crucial; priming with epigenetic therapy prior to chemotherapy may enhance susceptibility, whereas incorrect timing could elevate toxicity or activate alternative pathways. Therefore, combinations should be evaluated based on their mechanisms, scheduling, dosage, biomarkers, and supporting evidence [123].

#### 5.4.3. Combination with Targeted Agents

Epigenetic therapies can be integrated with targeted agents, including PARP inhibitors, anti-angiogenic therapies, and pathway-specific inhibitors. With PARP inhibitors, DNMT or HDAC inhibition may enhance susceptibility by suppressing homologous recombination, altering replication-fork protection, or reactivating silenced DNA-damage response mechanisms, depending on BRCA status and baseline repair competence. For anti-angiogenic therapy, the rationale is vascular normalization or disruption of hypoxia-driven epigenetic resistance. Epigenetic modulation may enhance the efficacy by modifying HIF-dependent transcription and further increasing tumor sensitivity to microenvironment-directed therapies [124].

#### 5.4.4. Timing, Sequencing, and Treatment Optimization

**Figure 3:**
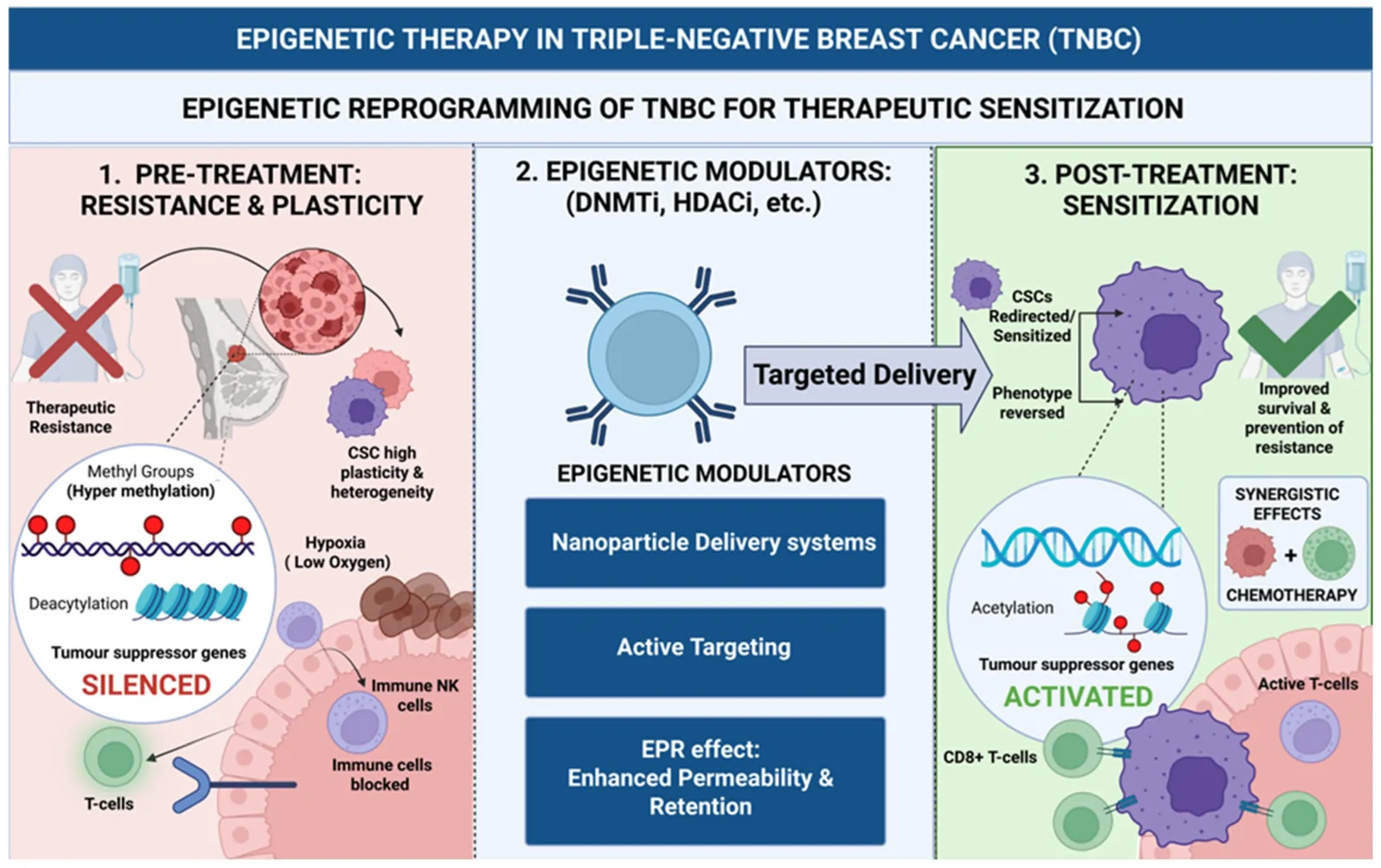
Epigenetic therapy reprograms TNBC to overcome therapeutic resistance. Epigenetic dysregulation in TNBC promotes tumor suppressor gene silencing, cancer stem-cell (CSC) plasticity, immune evasion, and treatment resistance. Epigenetic modulators, including DNA methyltransferase (DNMT) and histone deacetylase (HDAC) inhibitors, delivered through targeted nanocarrier systems, reverse these aberrant epigenetic programs. Reactivation of tumor suppressor genes restores apoptosis, reduces CSC-associated phenotypes, and enhances anti-tumor immune responses through increased T-cell activity. Consequently, epigenetic reprogramming sensitizes TNBC to conventional therapies, resulting in improved therapeutic efficacy and reduced resistance.

Treatment sequence may be as important as drug choice. Preclinical work suggests that epigenetic priming before chemotherapy or immunotherapy can create a transient window of increased susceptibility, whereas concurrent or prolonged exposure may increase toxicity without improving efficacy [125,126]. This principle is summarized in Figure 3, which illustrates epigenetic reprogramming of resistant TNBC states toward therapeutic sensitization.

### 5.5. Delivery and Toxicity Issues

While there have been positive signals from the initial studies and trials of TNBC, there remain significant challenges to the application of these therapies. Perhaps the biggest concern for the application of these therapies, as well as other epigenetic therapies, is their toxicity [127]. As discussed, agents such as DNMT inhibitors and HDAC inhibitors, as well as other epigenetic therapies, target the cancer cell by modifying the epigenome. This, as a positive effect, reprograms the cancer cell to respond to the drug. However, the same effect also impacts the normal cell, and as a result, there are often off-target effects. There are often hematologic toxicities, as well as non-hematologic toxicities, which include fatigue, GI symptoms, and cardiac events. While these effects are often dose-limiting and, for the most part, not completely reversible, they remain a significant concern for the application of these therapies [128].

The other significant obstacle to be overcome is that of tumor specificity. The fact that these drugs target chromatin-modifying enzymes that are present in cancer cells as well as normal cells makes them nonspecific. This nonspecificity might narrow the therapeutic index because it might affect normal cells in the body after a drug is administered [128]. For instance, DNMT inhibitors might alter gene expression in normal blood cells, and HDAC inhibitors might alter gene expression in heart or brain cells. Another complicating factor in this regard is that TNBC is a heterogeneous cancer type. This means that there are tumor cells within a tumor that might respond differently to these drugs [129]. Nanocarrier-based delivery may improve the therapeutic integration of epigenetic agents in TNBC. A targeted co-delivery system incorporating a DNMT inhibitor with chemotherapy enhanced tumor-directed treatment and immune modulation in preclinical TNBC models, supporting nanocarriers as an investigational strategy for improving combination efficacy while limiting nonspecific exposure [130].

Studies in preclinical models of TNBC using DNMT inhibitors and HDAC inhibitors in nanoparticle delivery have shown higher anti-tumor activity with fewer side effects compared with conventional formulations [131]. Apart from passive nanoparticle targeting, active targeting strategies are being investigated. Epigenetic compounds are being conjugated to tumor-targeting ligands, antibodies, or peptides, enabling them to target tumor-specific cell surface markers, such as folate receptor or EGFR, which are overexpressed in certain subtypes of TNBC. This method increases the local concentration of the epigenetic drug, which reduces its potential negative effects on the body as a whole [131,132].

Moreover, combining epigenetic agents with other active targeting methods, such as nanoparticles that release the drug based on pH, enzymes, and redox conditions, will improve the precision of targeting cancer cells. As a result, this strategy will not only make epigenetic compounds more effective but also reduce the chance of unwanted epigenetic changes in healthy tissues [133].

## 6. Enabling Technologies and Evidence Gaps

This section is restricted to technologies that can test, resolve, or stratify resistance mechanisms rather than to general TNBC innovation. Spatial multi-omic approaches can resolve malignant, stromal, and immune niches associated with therapeutic response [111], while CRISPR-based epigenome editing offers an experimental means to test whether candidate chromatin changes are causal [134].

Artificial intelligence and predictive modeling may help integrate multi-omic data for biomarker discovery and patient stratification, but their value depends on representative datasets, external validation, and clinically annotated outcomes [135].

These approaches remain enabling research platforms rather than established clinical tools. Single-cell and spatial studies are limited by tissue quality, sampling bias, batch effects, cost, and data integration; CRISPR approaches face delivery, off-target, and cell-type-specificity constraints; and AI models require transparent external validation and prospective testing before they can guide treatment decisions.

**Figure 4:**
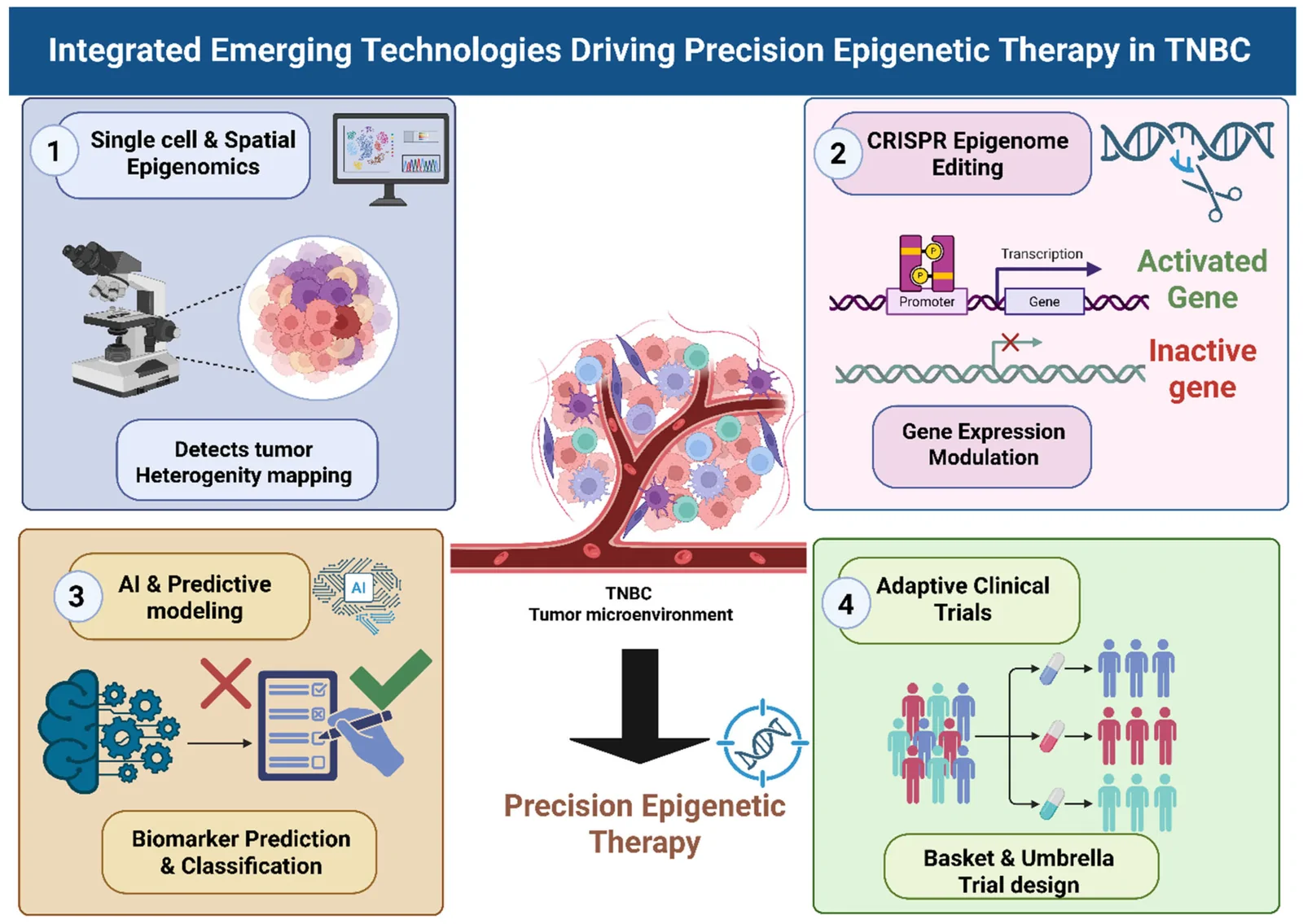
Enabling technologies for resistance-focused epigenetic research in TNBC. Single-cell and spatial profiling can resolve heterogeneous cellular states and multicellular niches; CRISPR-based epigenome editing can test causal regulatory mechanisms; artificial intelligence can integrate multi-omic data for biomarker discovery; and adaptive trial designs can evaluate biomarker-guided combinations. These approaches remain investigational and require validation before routine clinical use.

Figure 4 summarizes these enabling approaches. Their relevance to this review is limited to resistance-state discovery, causal validation, biomarker development, and biomarker-guided translational testing.

### 6.1. CRISPR Epigenome Editing

CRISPR-dCas9 epigenome editing can recruit DNA methylation or histone-modifying effectors to selected loci without altering the underlying DNA sequence. In TNBC, its present value is mechanistic: testing whether a candidate resistance-associated epigenetic state is causal and reversible. Therapeutic use remains experimental because delivery, off-target effects, cell-type specificity, durability, reversibility, and regulatory issues remain unresolved [134,136,137].

### 6.2. Artificial Intelligence and Predictive Modeling

Artificial intelligence and machine learning methods can integrate methylation, chromatin, transcriptomic, and clinical data to identify candidate resistance biomarkers and stratify patients. However, models require large, representative, well-annotated cohorts, external validation, transparent interpretation, and prospective testing before they can guide TNBC treatment decisions [135,138].

### 6.3. Clinical Translation Roadmap

Clinical translation should prioritize biomarker-defined enrollment, serial sampling, pharmacodynamic target-engagement measures, and trial designs that separate the effect of epigenetic priming from that of the partner therapy. Adaptive designs may be useful, but only when grounded in a validated biological marker and a prespecified treatment hypothesis [139,140].

## 7. Critical Synthesis and Conclusions

Epigenetic plasticity offers a coherent explanation for how TNBC cells can survive treatment without acquiring a single fixed resistance mutation. The strongest evidence supports reversible chromatin states associated with persister survival, epithelial–mesenchymal plasticity, altered DNA-damage repair, and immune escape; however, the evidence is uneven across mechanisms and often derives from cell lines, xenografts, or treatment-selected tumors.

Clinically, DNMT- and HDAC-directed combinations have demonstrated pharmacodynamic activity but not consistent efficacy. BET, EZH2, LSD1, and other demethylase-related vulnerabilities remain largely context-dependent and preclinical. The most plausible translational strategy is therefore not unselected epigenetic monotherapy, but biomarker-guided combinations in which treatment sequence, target engagement, and toxicity are measured explicitly.

Future studies should prioritize paired pretreatment and progression samples, longitudinal liquid-biopsy analysis, cell-resolved profiling, and prospective biomarker-defined trials. Epigenetic plasticity is a plausible therapeutic vulnerability, but its clinical value will depend on identifying the patients, cellular states, and treatment windows in which that vulnerability is selective, reversible, and durable.

## Figures and Tables

**Table 1:** Evidence appraisal of epigenetically mediated therapy-resistance mechanisms in triple-negative breast cancer.

Category	Representative Epigenetic Mechanism	Evidence Source	TNBC Specificity	Resistance Context	Clinical Maturity	Major Limitation	Ref.
General epigenetic plasticity	Reversible chromatin and DNA methylation states; non-coding-RNA regulation; drug-tolerant persister, EMT and metabolic-state transitions	TNBC cell lines, organoids and xenografts; broader cancer-plasticity studies	Moderate; not exclusive to TNBC	Multidrug tolerance and residual disease	Preclinical	Persister states lack standardized definitions and validated clinical markers	[5]
Chemotherapy resistance (drug efflux and apoptosis)	DNA methylation and histone modifications regulating ABCB1/MDR1 and pro-apoptotic proteins such as BIM and PUMA	Treatment-selected cell lines and xenograft models	Moderate; mechanisms also occur in other cancers	Anthracyclines, taxanes and platinum agents	Preclinical	Limited validation in paired pretreatment and post-resistance TNBC samples	[16,58]
Chemotherapy resistance (DNA-damage response)	Dynamic methylation of BRCA1 and other DNA-damage-response genes; compensatory repair-pathway activation	Tumor cohorts and mechanistic breast cancer models	Moderate–high for BRCA1; lower for MGMT	Platinum sensitivity and acquired resistance	Exploratory translational evidence	Methylation prevalence and functional consequences vary across assays and cohorts	[91,92]
Chemotherapy resistance (stemness and genomic instability)	ABCB1 activation, SOX2-associated H3K4me3 enrichment and LINE-1 hypomethylation	Resistant cell lines and observational tumor analyses	Moderate for SOX2 and ABCB1; low for LINE-1 as a resistance-specific marker	Doxorubicin and taxane resistance	Preclinical or exploratory biomarker stage	Associations do not consistently demonstrate epigenetic causality	[60,61,62]
PARP-inhibitor resistance (BRCA1 and homologous recombination)	Loss or reversal of BRCA1 promoter methylation, restoring BRCA1 expression and homologous recombination	PARP-inhibitor- or platinum-resistant models and patient-derived samples	High in BRCA1-methylated TNBC	Olaparib, talazoparib and platinum cross-resistance	Translational but not prospectively validated	Applicable only to tumors initially dependent on functionally significant BRCA1 methylation	[63,64,91,92]
PARP-inhibitor resistance (EZH2 and homologous recombination)	EZH2- and H3K27me3-associated regulation of homologous-recombination pathways, including RAD51-associated activity	Breast cancer and TNBC cell-line and xenograft studies	Moderate	PARP-inhibitor adaptation	Preclinical	Effects may differ according to BRCA status, treatment exposure and cellular context	[63,64,65]
PARP-inhibitor resistance (HDAC/PARG and chromatin regulation)	HDAC-dependent chromatin regulation, PARG silencing and HOTAIR-associated transcriptional remodeling	Mechanistic resistant-cell and xenograft studies	Low–moderate	Homologous-recombination restoration, replication-fork protection and reduced PARP trapping	Preclinical	Mechanisms are heterogeneous and rarely evaluated together in patient samples	[63,66]
PARP-inhibitor resistance (SWI/SNF and enhancer remodeling)	SWI/SNF disruption, H3K27ac remodeling, BET-protein activity and MYC-associated enhancer reprogramming	Genomic and functional studies in TNBC and other cancers	Moderate; dependent on the affected SWI/SNF component	Adaptive transcriptional persistence during targeted therapy	Preclinical	Limited validation prevents generalization to all SWI/SNF-deficient or ARID1A-altered tumors	[25,68]
Immune evasion and checkpoint resistance (MHC-I and interferon signaling)	Epigenetic repression of MHC-I components and interferon/JAK–STAT signaling	TNBC cell lines, mouse models and tumor-expression or methylation datasets	Moderate	Primary or adaptive resistance to PD-1/PD-L1 blockade	Preclinical and exploratory biomarker stage	Direct evidence from longitudinal immunotherapy-treated TNBC cohorts is limited	[62,69,70]
Immune evasion and checkpoint resistance (PD-L1 and antigen processing)	Context-dependent PD-L1 regulation; methylation of TAP1/LMP2; repressive chromatin states in tumor-associated macrophages	Breast cancer cell studies and immune–tumor preclinical models	Low–moderate; several findings are not TNBC-specific	Impaired antigen processing and checkpoint-inhibitor resistance	Preclinical	Direction of PD-L1 regulation varies by regulator, cell type and experimental model	[67,73,74]
Immune evasion and checkpoint resistance (STING/interferon and enhancer regulation)	BRD4-associated regulation of STING/interferon signaling, NEAT1 activity and PD-L1-associated enhancer remodeling	Primarily mechanistic cell-line and animal studies	Low–moderate	Immune escape and reduced checkpoint-inhibitor responsiveness	Preclinical	Sparse validation in paired human TNBC samples collected during immunotherapy	[76]
Metabolic reprogramming (glycolysis and lipid metabolism)	Epigenetic regulation of HK2, PKM2, CD36, LPL and ACC	TNBC metabolic experiments and resistant-cell models	Moderate	Chemotherapy tolerance and adaptation to nutrient stress	Preclinical	Metabolic remodeling may be a consequence rather than a cause of resistance	[77,78]
Metabolic reprogramming (metabolic-state switching and nutrient metabolism)	Switching between glycolysis and fatty-acid oxidation; histone lactylation; epigenetic regulation of glutamine and serine metabolism	TNBC models supplemented by broader cancer-metabolism evidence	Moderate overall; variable for individual pathways	Chemotherapy tolerance and possible immune resistance	Emerging preclinical evidence	Histone-lactylation and amino-acid-metabolism findings require further functional validation in TNBC	[77,78]
Microenvironment-driven resistance (hypoxia and CAF-associated remodeling)	Hypoxia/HIF-1α-associated HDAC and PRC2 activity; CAF-associated TET-dependent remodeling	Co-culture systems, animal models and tumor–microenvironment profiling	Moderate; pathways are shared across solid tumors	Immune exclusion and reduced treatment responsiveness	Preclinical	Bulk-tissue and co-culture studies provide limited cell-type-specific causal resolution	[86]
Microenvironment-driven resistance (macrophage and fibroblast signaling)	Macrophage and fibroblast chromatin remodeling involving EZH2, HDACs, TGF-β, HIF-1α and lactate signaling	Co-culture, murine and limited single-cell or spatial studies	Low–moderate	T-cell dysfunction and microenvironment-mediated therapeutic resistance	Preclinical	Predominant use of simplified M1-like/M2-like macrophage classifications, with limited validation in human TNBC	[70,89,90]

**Table 3:** Epigenetic therapeutic strategies in TNBC and their translational limitations, with evidence from broader breast cancer or non-TNBC models considered indirect where direct TNBC-specific validation is unavailable.

Drug Class	Representative Agents	Mechanism of Action	Potential Therapeutic Relevance in TNBC	Clinical Maturity	Implementation Challenges	Citations
DNA methyltransferase inhibitors (DNMTi)	Azacitidine, decitabine	Reduce DNA methylation through inhibition of DNA methyltransferases, potentially restoring the transcription of epigenetically silenced genes	May reactivate tumor-suppressive and immune-related programs and increase responsiveness to chemotherapy or immunotherapy	Early clinical; combination strategies have reached clinical testing, but TNBC-specific efficacy remains unestablished	Limited single-agent activity; optimal combinations, dosing schedules and predictive biomarkers remain uncertain	[101,102]
Histone deacetylase inhibitors (HDACi)	Vorinostat, panobinostat	Prevent histone deacetylation, increasing histone acetylation and altering chromatin accessibility and transcription	May promote apoptosis and enhance sensitivity to cytotoxic or targeted therapies in selected preclinical TNBC models	Clinical; HDAC inhibition has reached phase Ib/II testing in TNBC, with limited efficacy signals	Broad target activity, dose-limiting toxicity and the absence of validated patient-selection biomarkers	[101,102]
BET inhibitors	JQ1, OTX015	Disrupt the binding of BET proteins to acetylated chromatin, thereby modifying enhancer-dependent oncogenic transcription	Can suppress MYC-associated and other pro-survival transcriptional programs in experimental TNBC models	Preclinical in TNBC; TNBC-specific clinical validation remains limited	Adaptive resistance, toxicity and limited TNBC-specific clinical evidence	[6,115]
EZH2 inhibitors	Tazemetostat	Inhibit EZH2 methyltransferase activity and reduce H3K27 trimethylation, potentially reversing PRC2-mediated transcriptional repression	May restrict tumor growth or enhance treatment responsiveness in biologically selected TNBC models	Preclinical in TNBC; therapeutic activity remains dependent on molecular context and requires clinical validation	Effects vary according to molecular subtype, EZH2 function and the broader chromatin context	[5]

**Table 4:** Clinical studies directly evaluating epigenetic agents in TNBC or HER2-negative breast cancer cohorts containing TNBC.

Agent	Trial	Population	Main Result	Toxicity	Biomarker Strategy	Ref.
Entinostat with atezolizumab compared with placebo with atezolizumab	NCT02708680 (ENCORE 602), phase Ib/II	Previously treated advanced TNBC; *n* = 81	No significant PFS improvement; median PFS was 1.68 months compared with 1.51 months, and ORR was 10.0% compared with 2.4%	Greater treatment-related toxicity with entinostat; no improvement sufficient to offset the added toxicity	PD-L1 and immune-response correlatives	[118]
Decitabine followed by pembrolizumab and standard neoadjuvant therapy	NCT02957968, phase II window study	HER2-negative breast cancer; *n* = 46, including 28 TNBC and 18 HR-positive patients	Increased stromal TILs and PD-L1; 11 of 27 TNBC patients proceeding to surgery achieved pCR; the independent contribution of decitabine could not be determined	Adrenal insufficiency occurred in 13.0%, while rash and hypothyroidism occurred in 6.5% each	Paired biopsies, stromal TILs, PD-L1, and circulating monocytic MDSCs	[119]
Oral decitabine/cedazuridine (ASTX727) + paclitaxel + pembrolizumab	NCT05673200, phase I	Metastatic or unresectable TNBC; dose-finding and dose-expansion cohorts	Temporarily closed to accrual; safety/RP2D evaluation; efficacy not established	Adverse-event profile and dose-limiting toxicity are primary safety concerns	Serial blood sampling; baseline and on-treatment tumor biopsies in the dose-expansion cohort	[120]

## Data Availability

All data generated are present in the current manuscript.
